# Construction of a Phylogenetic Tree of Photosynthetic Prokaryotes Based on Average Similarities of Whole Genome Sequences

**DOI:** 10.1371/journal.pone.0070290

**Published:** 2013-07-26

**Authors:** Soichirou Satoh, Mamoru Mimuro, Ayumi Tanaka

**Affiliations:** 1 Graduate School of Life and Environmental Science, Kyoto Prefectural University, Kyoto, Japan; 2 Graduate School of Human and Environmental Studies, Kyoto University, Kyoto, Japan; 3 Institute of Low Temperature Science, Hokkaido University, Sapporo, Japan; 4 CREST, Japan Science and Technology Agency, Sapporo, Japan; University of Connecticut, United States of America

## Abstract

Phylogenetic trees have been constructed for a wide range of organisms using gene sequence information, especially through the identification of orthologous genes that have been vertically inherited. The number of available complete genome sequences is rapidly increasing, and many tools for construction of genome trees based on whole genome sequences have been proposed. However, development of a reasonable method of using complete genome sequences for construction of phylogenetic trees has not been established. We have developed a method for construction of phylogenetic trees based on the average sequence similarities of whole genome sequences. We used this method to examine the phylogeny of 115 photosynthetic prokaryotes, i.e., cyanobacteria, Chlorobi, proteobacteria, Chloroflexi, Firmicutes and nonphotosynthetic organisms including Archaea. Although the bootstrap values for the branching order of phyla were low, probably due to lateral gene transfer and saturated mutation, the obtained tree was largely consistent with the previously reported phylogenetic trees, indicating that this method is a robust alternative to traditional phylogenetic methods.

## Introduction

Construction of phylogenetic trees is important for understanding the evolutionary processes such as photosynthesis. Phylogenetic trees have been constructed using single genes; however, a large number of genes have been reported to be horizontally transferred among organisms [1], [2]. Therefore, conflicting tree topologies have resulted depending on the genes used for tree construction [3]–[5]. Another problem is saturation of nucleotide substitutions. In order to overcome these problems, concatenated sequences have been used for construction of phylogenetic trees. This approach, however, has limitations when distantly related organisms are analyzed, because the number of orthologous genes is limited. A new approach is needed: a phylogeny based on the whole genome sequence. This new approach is a potentially powerful tool for elucidating phylogenetic relationships.

A growing number of whole genome sequences has become available [6], enabling us to construct a phylogenetic tree using complete genome sequences [7]. Several methods of tree construction based on complete genome sequences have been proposed, such as gene order [8], gene content [9], nucleotide composition [10], metabolic pathway reaction content [11], and average sequence similarity [12]–[14]. The average sequence similarity approach utilizes whole genome sequences to represent the similarity between genomes. However, one method based on average sequence similarity excludes phylogenetically discordant genes, which exhibit different patterns of similarity from the majority of genes in the genome [12]. Therefore, the similarities between genomes are not sufficiently incorporated into an evolutionary distance matrix. In this sense, the methods for using complete genome sequences still need to be improved. A theoretical basis for taxonomic analysis using a whole genome approach has not been established [7], and resolution of taxonomic relationships has differed depending on the methods used [15]. Development of a reliable method based on whole genome sequences is absolutely necessary. In this study, we aimed to establish a new approach to phylogenetic analysis using the average sequence similarity of the whole genome.

In principle, distance-based phylogenetic trees are constructed by a distance matrix that is linearly related to a time-dependent phenomenon, such as substitution of nucleotides. Analysis of the sequence similarity of gene products (proteins) does not necessarily give an index that is linearly related to substitutions in gene sequences. Therefore, we developed a method to convert the similarity of amino acid sequences to a value corresponding to a nucleotide substitution rate in 16S rDNA. Based on this function, we further analyzed and constructed the phylogenetic trees of photosynthetic prokaryotes. There are many questions and interesting aspects concerning the evolution of photosynthesis and photosynthetic organisms. The evolution of photosynthetic prokaryotes may be continuous, but the evolution of photosynthetic systems from anaerobic to aerobic photosynthesis is clearly discontinuous [3], [16]. However, these processes are largely unknown.

There are six phyla of photosynthetic bacteria, Cyanobacteria, Proteobacteria, green sulfur bacteria (Chlorobi), green filamentous bacteria (Chloroflexi), Acidobacteria, and gram-positive bacteria (heliobacteria, Firmicutes), which are widely distributed in the eubacterial kingdom and a great number of their whole genome sequence are available. In this report, we improved the average sequence similarity method reported by Clarke [12] and adopted it to phylogenetic studies of photosynthetic prokaryotes.

## Materials and Methods

### BLAST Analysis

We calculated E-values using the blastp program version 2.2.16, [17] and modified Perl scripts, as in our previous report [18]. The deduced amino acid sequences of every gene from one organism were used as the query (query database) for a BLAST (Basic Local Alignment Search Tool) search against the protein database of another organism. For the calculation of E-values, we used default parameters and settings of BLAST as follows: a cut-off E-value of 10, the BLOSUM62 amino acid substitution matrix [19], and filtration of low complexity sequences [20]. E-values of the best-matched proteins, which showed the lowest E-values for each query sequence, were extracted. All E-values were converted into common logarithms, and E-values of zero were converted to −180 for data handling. These E-values were used for calculation of the evolutionary distances.

### Calculation of the Substitution Rate of 16S Ribosomal DNA

We independently calculated the substitution rate of 16S ribosomal DNA. The 16S rDNA sequences were retrieved from the website databases of DDBJ/EMBL/GenBank, CyanoBase (Kazusa DNA Research Institute), and Integrated Genomics Inc., and the database in the ARB software 7.7.12 [21]. Accession numbers of 16S rDNA genes and databases for retrieving 16S rDNA sequences are listed in Table S1. Pairs of 16S rDNA nucleotide sequences from two different organisms were aligned using CLUSTALW 1.81 with an IUB matrix [22]. Substitution rates were calculated for all combinations of 16S rDNAs in all organisms used in this study even though some organisms contain multiple copies of 16S rDNA genes. The regression curve between the results based on the E-value estimation and the substitution rate of 16S ribosomal DNA was estimated using the IGOR Pro software (Version 5.05J, WaveMetrics, Inc. USA), and a correlation coefficient of determination was obtained using Microsoft Excel.

### Phylogenetic Analysis

The phylogenetic tree was constructed as a neighbor joining (NJ) tree [23] with the program NEIGHBOR from the PHYLIP package 3.67 [24]. The consensus NJ tree was constructed with the programs NEIGHBOR and CONSENSE from the PHYLIP package 3.67. Bootstrap values were constructed using the CONSENSE program [24] from 100 reproduced trees. Reproduced trees were formed from individual distance matrices that were constructed by randomly extracted best-matched proteins and their E-values. The rand function subprogram of the Perl language was used to select the best-matched proteins and their E-values.

We constructed a phylogenetic NJ tree based on 16S rDNA sequences using 1,364 unambiguously aligned bases to compare a branching pattern with those based on amino acid sequences. The distance matrix and phylogenetic tree were constructed using DNADIST with the Jukes-Cantor correction [25] and NEIGHBOR in the PHYLIP package 3.67, respectively. Bootstrap analysis of 100 replicates of the trees was performed with SEQBOOT, DNADIST and NEIGHBOR from the PHYLIP package. The CONSENSE program was used to obtain the bootstrap values. *Synechococcus elongatus* PCC 6301 were used as an out-group because only *Prochlorococcus* and *Synechococcus* groups were analyzed.

### Protein Sequence Databases

FASTA-formatted sequence files for whole proteins of each organism were retrieved from DDBJ/EMBL/GenBank, CyanoBase, Department of Energy Joint Genome Institute (JGI), Cyanorak database (http://www.sb-roscoff.fr/Phyto/cyanorak/), and Integrated Genomics Inc., and their sources are listed in Table S2.

## Results and Discussion

### Estimation of Evolutionary Distance Using a Two-dimensional Matrix

In construction of phylogenetic trees, the topology and branch length of the trees are estimated from a distance matrix. Therefore, it is necessary to obtain a distance matrix by the comparison of whole genomes when using the average sequence similarity method. We first compared whole protein sequences of two genomes by BLAST (Basic Local Alignment Search Tool), and plotted E-values of all best-matched pairs on a two-dimensional matrix. We then estimated the similarity between the two genomes using the averaged coordinate values of the data points on the two-dimensional matrix. We compared the similarity of the whole genomes with the substitution rate of the corresponding 16S rDNAs, and obtained a correlation equation for the relationship between the genome similarity and the nucleotide substitution rates.

**Figure 1:**
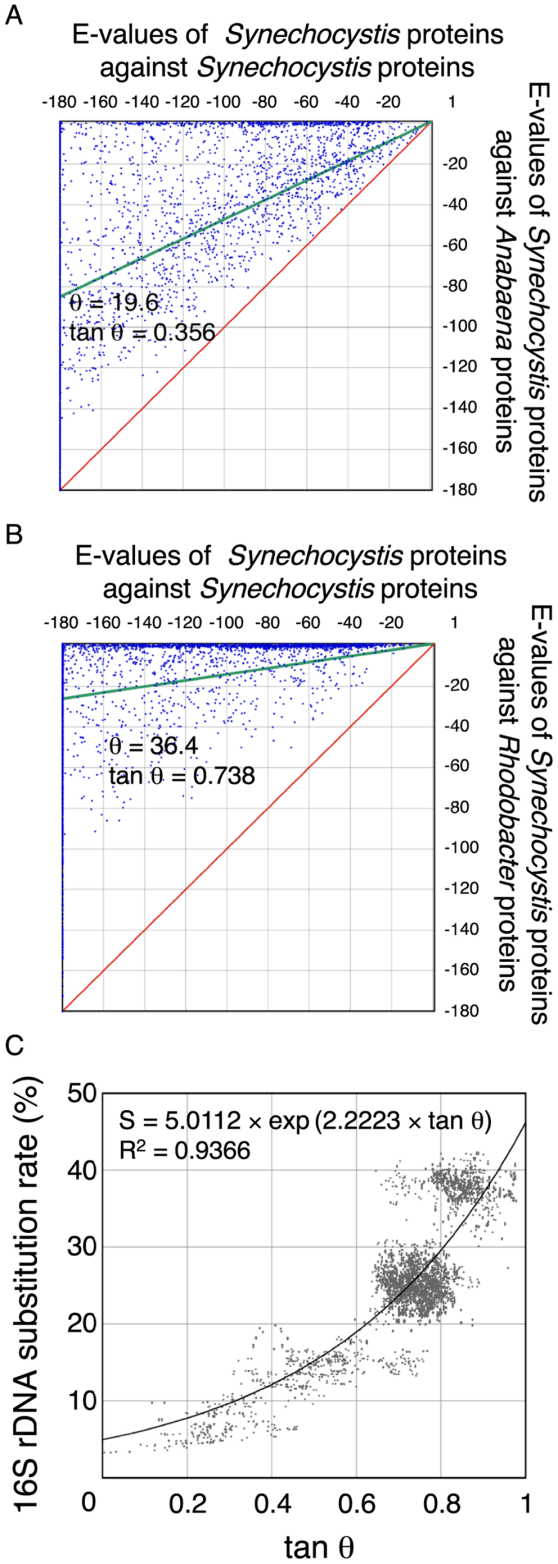
Evolutionary distance based on protein similarity and its relationship to rDNA substitution rate. Representation of best-matched proteins on a two-dimensional display. The vertical axes represent the logarithmic E-values of the best-matched proteins of *Anabaena* sp. PCC 7120 (A) and *Rhodobacter sphaeroides* 2.4.1 (B) to the proteins of *Synechocystis* sp. PCC 6803. The horizontal axes represent the logarithmic E-values of the best-matched proteins of *Synechocystis* sp. PCC 6803 to *Synechocystis* sp. PCC 6803 (A, B). In this case, the best-matched proteins are identical to the query proteins. The green lines are linear regression lines and the red lines mark the diagonal. θ is the angle between the red and green lines. (C) Relationship between tan θ and the 16S rDNA substitution rate. Each point represents the tan θ values (vertical axis) calculated with two genomes and the substitution rates of their 16S rDNA sequences (horizontal axis). The solid line represents the regression curve.

As a functional example of the method used for estimating similarity between species, all deduced amino acid sequences of *Synechocystis* sp. PCC 6803 (hereafter referred to as *Synechocystis*) were used as a query. When all the deduced amino acid sequences of *Synechocystis* were used as a query database for BLAST analysis against the deduced protein sequence database of the whole *Synechocystis* genome, E-values were primarily dependent on the lengths of individual query proteins. When these values were plotted on a two-dimensional display where the two axes are the E-values of *Synechocystis* proteins against *Synechocystis* protein database with a logarithmic scale, all values fell on the diagonal (data not shown). In the second step, the best-matched scores of *Synechocystis* were calculated against *Anabaena* sp. PCC 7120 (hereafter referred to as *Anabaena*) and plotted on a two-dimensional matrix where one axis represents the *Synechocystis*-*Synechocystis* pair and the other the *Synechocystis*-*Anabaena* pair. This revealed that almost all data points were localized near the axis of the *Synechocystis*-*Synechocystis* pair, because E-values were much smaller when comparing identical databases (Figure 1A). However, each data point plotted on the two-dimensional matrix includes the alignment length relative to query sequence, and the similarity in the aligned regions, because these points exhibit E-values against the best-matched proteins with the reference E-values against the identical proteins (Fig. 1A).

When the same calculations were performed on *Synechocystis* and the photosynthetic bacterium *Rhodobacter sphaeroides* 2.4.1, the data points were primarily localized in the area close to the *Synechocystis*-*Synechocystis* axis, because the similarities between *Rhodobacter* proteins to *Synechocystis* proteins were very low (Fig. 1B). These data were consistent with the current interpretations of the evolutionary relationships among photosynthetic prokaryotes [26].

In order to numerically express the distribution of individual points on the two-dimensional matrices, we obtained the following two values (AvE_X_, AvE_Y_) by the averaging of coordinates of the data points.
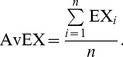
(1)

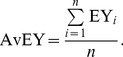
(2)Where ΣE_Xi_ and ΣE_Yi_ are numerical sums of the X- and Y-coordinate values, respectively, and n stands for the number of data points.

From these two values, we calculated the similarity (m) between two genomes as follows.
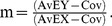
(3)Where C_OV_ is the cut-off value for BLAST calculation, and C_OV_ was subtracted from AvE_X_ and AvE_Y_. This manipulation was introduced to move the origin of the scatter plot from the point (C_OV_, C_OV_) to the point (0, 0). The slope of the green lines in Figures 1A and 1B equals m. Theoretically, m is equal to 1 when the two genomes are identical, and m is equal to 0 when the two genomes have no similarity. The difference between the green line and the diagonal (m = 1) was calculated by the following equation.



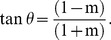
(4)As shown in Figures 1A and 1B, the closer the phylogenetic relationship, the smaller the value of tan θ. When two genomes are closely related, tan θ is close to 0; in contrast, when the two genomes are distantly related, tan θ is close to 1, indicating that tan θ reflects the averaged similarity of all best-matched pairs between the two genomes.

The tan θ value is an index for estimation of the phylogenetic relationship between two organisms. When this index is linearly related to the substitution rate of amino acids or nucleotides in the genomes, it can be used as a phylogenetic distance. 16S rDNA gives a reliable marker sequence for evolutionary analysis due to its universal distribution, sufficient size, and variability of sequence [27]. The average number of nucleotide substitutions per 16S rDNA site represents the evolutionary distance. The substitution rate of 16S rDNA sequences was calculated using the following equation.
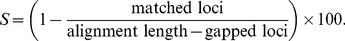
(5)Where S is the substitution rate of 16S rDNA (%). The average numbers of nucleotide substitutions per site in 16S rDNA, listed in Table S1, were calculated from sequence alignments of 16S rDNAs without gaps.

We examined the relationship between tan θ and 16S rDNA substitution rates (S) in the combinations of 55 species, as shown in Fig. 1C. The total number of spots was 5,482, which corresponded to the combination of the substitution rates of 16S rDNAs and tan θ (Tables S1 and S2). Each pair of genomes has multiple spots on the graph because (i) a fraction of the organisms have multiple 16S rDNA genes in their genomes (Table S1), and (ii) two values of tan θ were obtained due to the interchange of query and reference databases. These two values of tan θ are usually different, due to the difference in gene composition between the two whole genome databases, indicating that the BLAST results are asymmetric [14]. This asymmetry gives rise to variations of data points for regression analysis. However, it is not reasonable to select only one of the two tan θ values for the regression analysis. We used all data points for the regression analysis. The two indices, tan θ and S, were not linearly correlated; however, we were able to obtain an exponential regression line as follows:
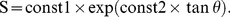
(6)


The two constants were estimated as follows: const1 = 5.0112 and const2 = 2.2223. The correlation coefficient (R^2^) of this equation was 0.9366, high enough to enable further estimation of distances. This equation shows that the similarity between two genomes (tan θ) can be converted to an index that is linearly correlated with the time-dependent phenomenon, i.e., the substitution rates of the 16S rDNA sequence (S). One tan θ value gives rise to a unique S value; thus, tan θ can be used for construction of phylogenetic trees after conversion to an S value.

The blastp-based distance method has already been reported previously [12], [13]; however, the present method differed in the following points. First, a new procedure for normalization of the alignment score was used. Since an alignment score of the best-matched pair depends on the length of the genes compared, correction of the length-dependent property is required. In previous reports [12], [13], an alignment score of the best-matched pair calculated by BLAST was divided by an alignment score of the query sequences when the identical sequence was used as a query to generate a normalized score. Subsequently, the average of all the normalized scores was calculated. However, normalized scores of short sequences (corresponding to small proteins) were over-estimated in this method, because the alignment length of each protein was excluded from the calculation of the averaged score; therefore, similarities of various lengths of alignment were evaluated for the calculation of averaged score independent of lengths. In contrast, in this study, the best-matched E-values corresponding to alignment scores of the best-matched pairs were first converted to a logarithmic scale and averaged, and the resultant values were divided by the averaged value of the E-values in a logarithmic scale of the identical proteins (see equation 3). Our normalization procedure properly represents the whole genome similarity, because small proteins are not over-estimated, contrary to previous reports [12], [13].

The second difference is in selection of genes for the calculation of evolutionary distance. In a previous report [12], [13], specific genes, whose orthologous genes (bidirectional best-match procedure) exist in at least four other genomes, were selectively used, leading to exclusion of a significant fraction of genes. The excluded fraction was estimated to be in the range of 6 to 69% of the total genes when the genomes of 28 bacteria, 8 archaea, and 1 eukaryote were used for the analysis [12], [13]. In our method, almost all the protein sequences were used for the calculation, indicating that the evolutionary distances calculated in this report properly reflected the whole genome similarity.

### Construction and Evaluation of a Phylogenetic Tree Based on Whole Genome Comparisons within a Specific Phylum

**Figure 2:**
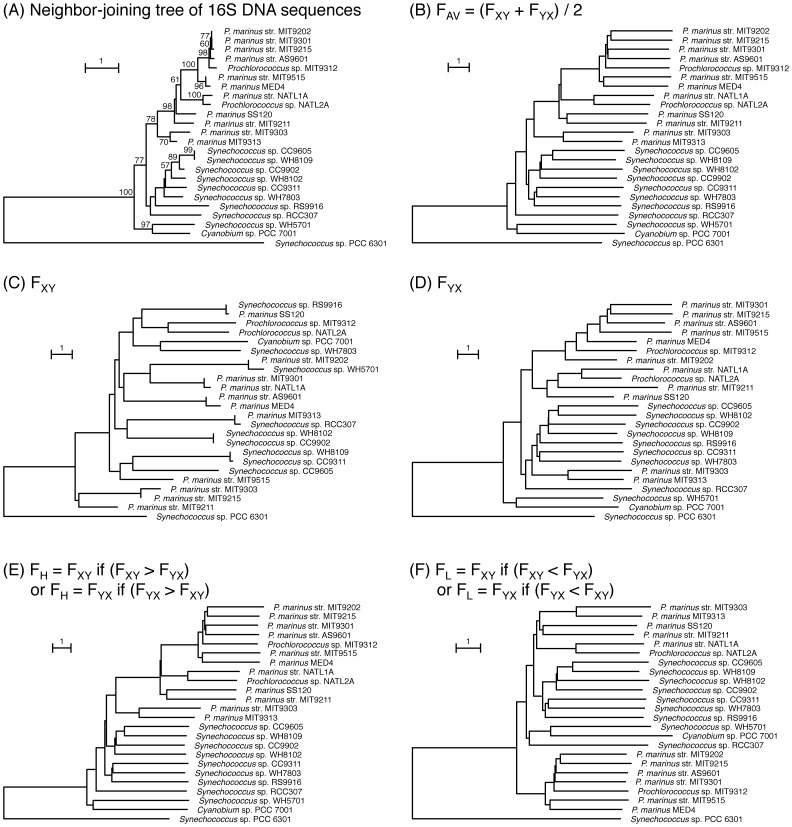
Phylogenetic trees of *Prochlorococcus* and *Synechococcus* species. Phylogenetic tree of 16S rDNA sequences (A). The lengths of the nodes represent the substitution rate, which is defined as the percentage of substitution sites per alignment length. Bootstrap values ≥50 are shown on the branch points. (B) to (F). Phylogenetic trees were constructed using F_Av_ (B), F_XY_ (C), F_YX_ (D), F_H_ (E) and F_L_ (F) values. The lengths of the nodes in the trees (B) to (F) represent the F_Av_, F_XY_, F_YX_, F_H_, and F_L_, respectively. Phylogenetic trees were drawn as NJ trees using the NEIGHBOR program in the PHYLIP package 3.67. Out-group of phylogenetic trees is the same as in (A).

We constructed a phylogenetic tree using a specific clade within one phylum to evaluate the validity of our method. We used the whole genome databases of the marine cyanobacteria *Prochlorococcus* and *Synechococcus* because the whole genome sequences of many organisms in this clade are available, and their evolutionary relationships have been extensively studied [28]. In spite of the close relationship among these organisms, the genome size and gene content are very different, especially in the *Prochlorococcus* lineage (1.66 Mbp for *Prochlorococcus marinus* MED4 and 2.61 Mbp for *Synechococcus* sp. CC9311 (Table S3)). Furthermore, lateral gene transfer is reported to have occurred frequently among these organisms [29], [30]. The tree constructed using 16S rDNA demonstrated the evolutionary relationships between *Prochlorococcus* and *Synechococcus* [31] (Fig. 2A). Based on these observations, we evaluated the effect of gene content and lateral gene transfer on the construction of our tree.

**Figure 3:**
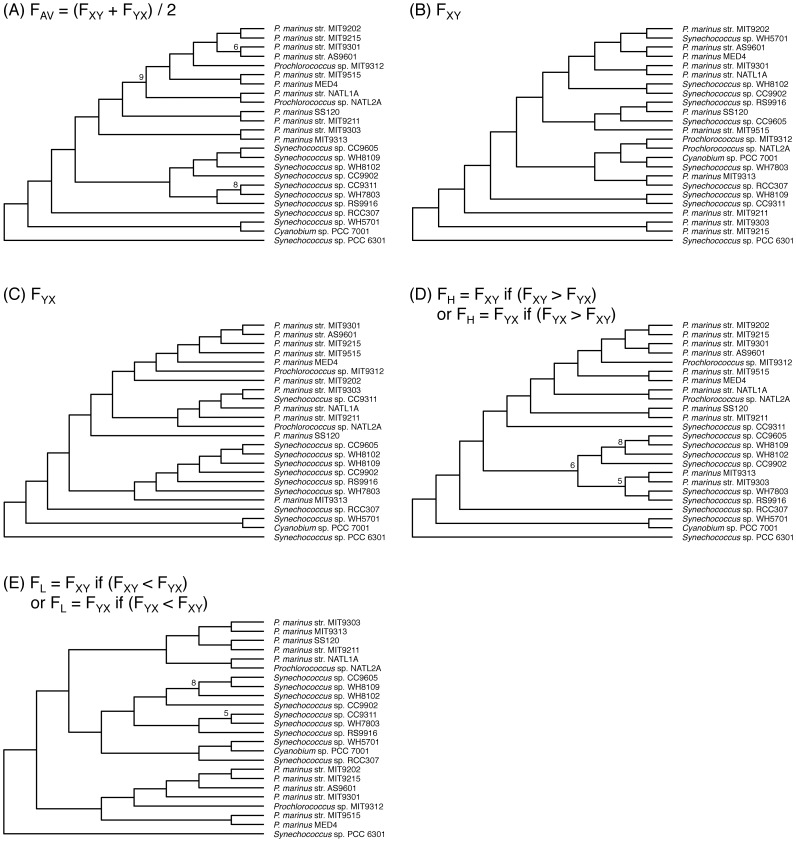
Phylogenetic tree constructed using a reduced gene number for the *Synechococcus* sp. CC9311 genome. Ten independent databases of *Synechococcus* sp. CC9311 were artificially formed with 289 randomly selected genes (10% of the total gene number). Ten independent phylogenetic trees using five *Prochlorococcus* species and four *Synechococcus* species containing artificially formed *Synechococcus* sp. CC9311 databases were constructed using each of the distance indices used to generate Figs. 2B to 2F. A consensus tree for the ten independent trees was generated with the use of the CONSENSE program for each of the five distance indices, F_Av_, F_XY_, F_YX_, F_H_, and F_L_. Numbers on the branch points represent the number of identical branching patterns in ten independent trees. Branching points without numbers indicate that the number of identical branching patterns is ten. *S. elongatus* PCC 6301 was used as an out-group.

Upon construction of trees, asymmetric effects cannot be avoided when two organisms are used. To escape this asymmetric effect, several approaches have been adopted. One of these is an average value of the two distances depending on the two queries [14]. We examined the asymmetric effect on the branching patterns of phylogenetic trees that were constructed by distance matrices defined by different indices (Figs. 2, 3 and S1).

We defined the index F, which corresponds to the S value as a function of tan θ. When F was calculated using the whole genome databases X and Y, we defined F as F_XY_, which denotes the value for the whole genome database X used as a query against the whole genome database Y. F_XY_ corresponds to tan θ_XY_ through S. Accordingly, F_YX_ is not identical to F_XY_, because tan θ_YX_ is different from tan θ_XY_. This procedure can be regarded as finding a data point S on the tan θ-S regression line (Fig. 1C). We further defined the following indices as the values of F for distance calculations.
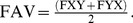
(7)

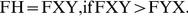
(8a)

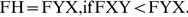
(8b)

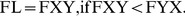
(9a)

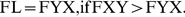
(9b)


Fig. 2 shows the phylogenetic trees constructed using various indices of F, as defined by equations 7 to 9. The tree constructed using the 16S rDNA sequences (Fig. 2A) is shown for reference, with *Synechococcus elongatus* PCC 6301 as out-group. In this tree, 11 species of *Synechococcus* and 13 species of *Prochlorococcus* were clearly separated into two clades. The topologies of the trees using F_AV_ (Fig. 2B) and F_H_ (Fig. 2E) were similar to that of the 16S rDNA tree and almost identical to that by Large-Scale Phylogenomic Analyses [32]. On the contrary the tree by F_XY_ and its topology was largely different from that of the 16S rDNA (Fig. 2C). Two *Prochlorococcus* species belonged to the *Synechococcus* cluster (Fig. 2D). In the tree constructed using F_L_ (Fig. 2F), *Prochlorococcus* most deeply branched. These results indicate that the trees using the F_AV_ (Fig. 2B) and F_H_ (Fig. 2E) indices gave rise to the reasonable branching pattern.

Gene content could potentially affect the topology of the phylogenetic trees. We constructed trees using the same data sets as in Fig. 2, but with the exception that the gene content for one organism was reduced, i.e., the gene content of *Synechococcus* sp. CC9311 or *Synechococcus* sp. WH8102 was reduced to 10% of the total by random selection (Figs. 3 and S1). When the branching orders were compared, trees using the F_XY_ (Fig. 3B), F_YX_ (Fig. 3C)_,_ F_H_ (Fig. 3D), and F_L_ (Fig. 3E) indices gave rise to branching orders different from those constructed using the total number of genes (compare Figs. 2C to 2F with Figs. 3B to 3E) and *Prochlorococcus* did not form a single cluster. In contrast, the topology of the tree using F_AV_ was not affected by the reduction in genome size (Fig. 2B vs. Fig. 3A). Identical tendencies were obtained with other data sets in which the gene content of *Synechococcus* sp. WH8102 was reduced to 10% of the original (Fig. S1). Thus, only the tree drawn using the F_AV_ index was not affected by the gene content. Symmetric differences, which represent an index of similarities of tree topologies [33], among various consensus trees (Figs. 2, 3 and S1) also indicated that the topologies of the F_AV_-based trees alone did not vary among trees (data not shown). These results clearly indicated that F_AV_ is a suitable index for calculation of distances. Hereafter, we constructed phylogenetic trees using F_AV_ as an index irrespective of gene content.

Additivity of an evolutionary distance is a very critical property for the construction of a reliable phylogenetic tree. We examined the additivity of the F_AV_ index. The Fitch-Margoliash (FM) tree is drawn using an additive tree method [34], assuming that distances along a tree are additive. An FM tree of *Prochlorococcus* and marine *Synechococcus* species constructed using F_AV_ (data not shown) was consistent with the NJ tree shown in Fig. 2B. The correlation coefficient between the branch lengths in the FM tree and the F_AV_ in the distance matrix was 0.9977. This high correlation coefficient strongly suggests that the F_AV_ is a suitable index for additivity of distance. Before a large scale of analysis, we constructed phylogenetic trees of a small number of species (55 species) based on F_AV_ (Fig. S2) and on the F_AV_ estimated by reciprocal best BLAST hits (Fig. S3). According to the tree by the reciprocal best BLAST hits, marine type *Synechococcus* clade branched off from the radiation of *Prochlorococcus*, and *Gloeobacter violaceus* PCC 7421 did not branch off most deeply, which are different from the supported topology of the phylogenetic tree of cyanobacteria [34]–[36]. These problems are solved in the tree constructed by F_AV_. Therefore, we defined F_AV_ as a distance for the construction of phylogenetic trees.

We also used the FM method for the construction of a phylogenetic tree to validate the NJ tree, because the FM method is one of the statistically sophisticated methods for the construction of a distance-based phylogenetic tree. Although comparison of the NJ and FM trees showed incongruencies of the branching pattern in some internal branches of cyanobacteria and purple bacteria and the branching position of the clade of green filamentous bacteria, the topologies of these two trees were still almost identical (data not shown). Comparison of the two trees suggests that the distance matrix constructed by our method produced almost completely congruent results, even though two distinct methods were used for the construction of the trees. Therefore, we adopted the NJ tree for comparison of our phylogenetic tree with other reported phylogenetic trees.

### Construction and Evaluation of a Phylogenetic Tree of Photosynthetic Prokaryotes Based on Whole Genome Comparisons

**Figure 4:**
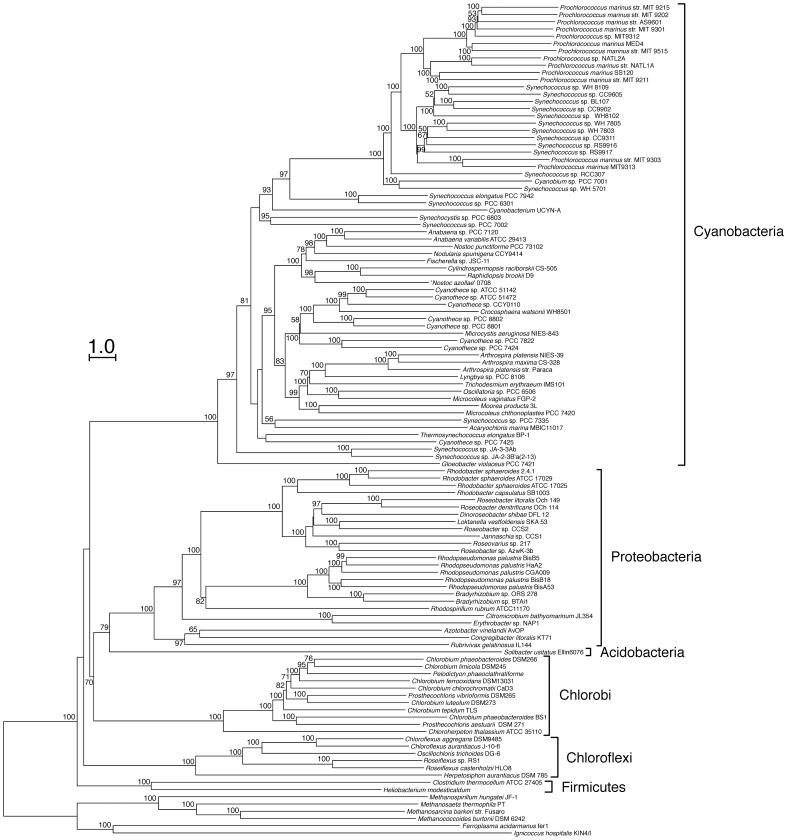
Phylogenetic tree of photosynthetic prokaryotes based on the average sequence similarity. Procedures for construction of the phylogenetic tree are the same as in Figs. 2B and S2. Bootstrap values ≥50 are shown on the branch points. Bootstrap values were obtained from 100 reproduced trees of 1,000 randomly selected E-values as all genomes contain more than 1000 genes. Archaea were used as an out-group.

We constructed phylogenetic trees of photosynthetic prokaryotes using the NJ method and the F_AV_ distance (Fig. 4), and evaluated the validity of our method in the Eubacteria kingdom by inspection of branching patterns. We used 115 photosynthetic organisms from five phyla: cyanobacteria, proteobacteria, green sulfur bacteria (Chlorobi), green filamentous bacteria (Chloroflexi) and gram-positive bacteria (Firmicutes). Archaea and some non-photosynthetic bacteria were also included for consideration. We initially inspected the branching pattern in each phylum, and then examined the branching pattern among phyla.

Inspection of the branching patterns in cyanobacteria revealed the following features. Within the cyanobacterial clade, *Gloeobacter violaceus* PCC 7421 branched off most deeply followed by the branching of *Synechococcus* sp. JA-3-3Ab and *Synechococcus* sp. JA-2–3B’a(2–13). The other cyanobacteria were divided into two groups. The first group consisted of *Prochlorococcus* and *Synechococcus*, and the second group contained all other cyanobacteria. These cyanobacterial branches are consistent with previous reports [32], [35], [36] with the exception of *Synechocystis* sp. PCC6803 and *Synechococcus* sp. PCC7002. These two organisms branched off at the root of the second group and the bootstrap value for the branching was low.

Our tree showed that the 10 *Synechococcus* species and 11 *Prochlorococcus* species were classified into two clades, with the exception of *P. marinus* MIT9313 and *P. marinus* str. MIT9303, which were assigned to the *Synechococcus* clade (Fig. 4). The *Prochlorococcus* species include two major ecotypes [37], [38]: high-light-adapted and low-light-adapted species that are distributed in different water columns in the ocean. The present tree showed that a high-light-adapted species branched off from a low-light-adapted species after the low-light-adapted species branched off from other *Synechococcus* species. The branching pattern of these two ecotypes was consistent with that of the phylogenetic tree based on 16S rDNA [31] and other genes [35], [36]. In the present tree, *P. marinus* MIT9313 forms a single cluster within the *Synechococcus* species. The branching pattern of *P. marinus* MIT9313 was determined by its F_AV_ (Table S4). The F_AV_ between *P. marinus* MIT9313 and *Synechococcus* species was smaller than the F_AV_ between *P. marinus* MIT9313 and other *Prochlorococcus* species. Conflicting results have been reported concerning the relationship of *P. marinus* MIT9313 to other marine cyanobacteria [31], [35], [36]. A phylogenetic tree based on the concatenation of 323 core proteins was consistent with the present tree [35]. In contrast, phylogenetic trees based on 16S rDNA sequences and 848 concatenated protein families support the presence of two sub-clades of *Prochlorococcus* and *Synechococcus* [31], [36]. These results suggest that similarities among most of the structural genes between *P. marinus* MIT9313 and *Synechococcus* species are slightly higher than that of 16S rDNA.

Purple bacteria belong to the proteobacteria. We demonstrated that proteobacteria were divided into three clades (α-proteobacteria, β-proteobacteria *Rubrivivax gelatinosus*, γ-proteobacteria *Azotobacter vinelandii* AvOP and *Congregibacter litoralis* KT71). Purple bacteria belonging to the clade of α-proteobacteria, were divided into two sub-clades (Fig. 4). One sub-clade consisted of *Rhodobacter* and *Roseobacter* species. The second sub-clade included *Rhodospirillum rubrum* ATCC 11170, *Bradyrhizobium* and *Rhodopseudomonas* species. The branching pattern of these species was consistent with that in phylogenetic trees based on the 16S rDNA sequences and the concatenation of alignments for 104 protein families [39], [40].

Branching patterns of green sulfur bacteria in the present tree were consistent with those based on the sequences of 16S rDNA, the gene encoding the Fenna-Matthews-Olsen (FMO) protein, and other proteins [41]–[43], and *Chloroherpeton thalassium* ATCC 35110 branched most deeply [44]. Previous studies have shown that the green sulfur bacteria were classified into four groups that were very closely related to each other [41], [43]. This classification differed from the species classifications, and showed that some species belonged to plural groups. *Prosthecochloris aestuarii* DSM271 is a marine bacterium and is included in the group 1 strains. The group 2 strains form vibrio-shaped cells, require a low salt concentration, and include *Chlorobium luteolum* DSM273 and *Prosthecochloris vibrioformis* DSM265. Group 3 strains form rod-shaped cells and are freshwater bacteria, and include *Chlorobium phaeobacteroides* DSM266, *Chlorobium limicola* DSM245, *Pelodictyon phaeoclathratiforme* and *Chlorobium ferrooxidans* DSM13031. Group 4 includes both freshwater and low salt strains; *Chlorobium tepidum* TLS is included in this group. In this classification, groups 2 and 3 were closely related, and groups 1 and 4 were distantly related to the other groups. The present tree showed that the group 1 and 4 strains diverged first in the cluster of green sulfur bacteria, and the strains of groups 2 and 3 branched off from the group 4 strains, and were closely related (Fig. 4). The branching pattern of these groups were identical to the phylogenetic trees based on 16S rDNA, the *fmoA* gene, and concatenated sequences for 12 highly conserved proteins [41]–[43]. Our results indicate that a phylogenetic tree constructed using whole genome sequences could reproduce the previously accepted classification of green sulfur bacteria based on the phylogenies of 16S rDNA and *fmoA* [41], [43].

The green filamentous bacteria clade containing *Roseiflexus* and *Chloroflexus* was divided into sub-groups in the present tree (Fig. 4), and this branching pattern was consistent with a previous report [45].

Based on the branching pattern and branching order of species within each phylum, we have demonstrated that our tree, based on whole genome sequences, is congruent with the previously reported classifications and phylogenetic trees based on 16S rDNA and other conserved genes. We found some differences in branching patterns of closely related organisms between our tree and phylogenetic trees based on single genes [31], [41].

The branching order of phyla (Fig. 4) was consistent with that of the tree based on the 102 orthologous proteins with mesophilic archaea as the out-group [46] and that on the comparison of feature frequency profiles of whole proteomes [47]. In contrast, the present tree was not congruent with trees based on the cytochrome *bc-*complexes [48], proteins involved in chlorophyll biosynthesis [49] and 16S rDNA [26]. Different branching patterns of phyla were reconstructed when the trees were constructed with different set of species (compare Fig. 4 and Fig. S2). These discrepancies might be due to horizontal gene transfer, in addition to biases in sequence change and large evolutionary distances. In fact, genome comparison shows that a significant number of cyanobacterial proteins from photosystems, photosynthetic electron transport, the inorganic carbon concentrating apparatus and chlorophyll biosynthesis were more similar to the proteins of green filamentous bacteria, green sulfur bacteria or heliobacteria than to those of purple bacteria (Fig. S4), indicating that the evolution of the photosynthetic machinery was accompanied by lateral gene transfer [3], [50].

### Taxonomic Resolution

**Figure 5:**
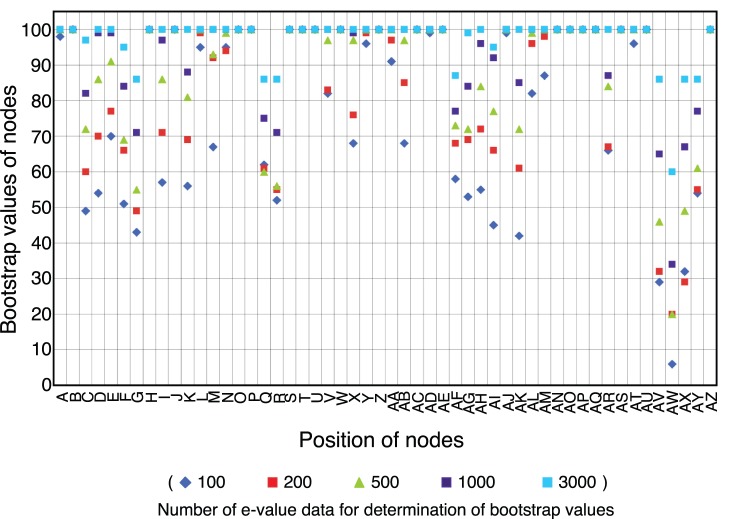
Relationship between the bootstrap values and the numbers of best-matched pairs. The bootstrap values of the nodes were determined using 100 reproduced trees with various amounts of best-matched pairs. Alphabetical characters of the branch points were represented in Fig. S2. Blue diamond, 100 E-values; red square, 200 E-values; yellow-green triangle, 500 E-values; purple square, 1,000 E-values; light-blue square, 3,000 E-values.

The reproducibility of the branching pattern constructed by our method should be evaluated by a statistical index. For this purpose, we introduced bootstrap values to our phylogenetic tree. Based on the phylogenetic tree of 55 species (Fig. S2), we calculated the evolutionary distances using 100 to 3,000 randomly selected, best-matched pairs, allowing overlapping selections, and constructed 100 independent trees (Fig. 5). Based on these trees, we estimated the bootstrap values for every node, a measure equivalent to the bootstrap values used in the phylogenetic trees constructed by other methods. Fig. 5 shows that the bootstrap values of all nodes within one genus (Fig. S2) were higher than 70 when 500 genes were used (nodes H-J, K-M, T-V, AF-AN, AO, and AP in Fig. 5). When 1,000 genes were used for the analysis, bootstrap values of all nodes within phyla were higher than 70 (nodes A-S, T-AD, AE, AF-AN, and AO-AQ in Fig. 5). Upon estimation of the branching order in cyanobacteria, two points that have been previously reported were unfavorable for analysis. First, cyanobacteria have acquired 9.5–16.6% [1] or more [2] of the genes in their genomes by lateral gene transfer. Secondly, genome size and gene content differ widely among the cyanobacteria: the smallest genome is 1.7 Mbp for *Prochlorococcus marinus* MED4 and the largest is 8.2 Mbp for *Nostoc punctiforme* PCC 73102 (Table S2). However, lateral gene transfer and gene content did not dramatically affect the tree topology within phyla because bootstrap values higher than 70 were obtained when 1,000 or more genes were used. Furthermore, bootstrap values of the nodes corresponding to the branching points between phyla (AV, AW and AX in Fig. 5) were lower than 70 when 1,000 genes were used. However, these branch points showed sufficiently high bootstrap values when 3,000 genes were used. These results indicate that the gene number required for reliable tree topology is variable among phylogenetic hierarchies, and strongly suggest that the gene content of cyanobacteria and photosynthetic bacteria is sufficient for inferring phylogenetic relationships. Bootstrap values of the nodes of deeply branched taxa were not sufficiently high, but this result may reflect the fact that phylogenetic resolution in deeply branched taxa was insufficient.

In summary, we have developed an improved method for the construction of phylogenetic trees based on the average sequence similarity of whole genomes. We applied this method to photosynthetic prokaryotes for the first time (Fig. 4). Although the phylogenetic relationships inferred from whole genome sequences were almost congruent with that of 16S rDNA and conserved orthologous genes, our tree differed slightly from trees based on single gene comparisons especially for the closely related organisms. Although rDNA is a good marker for phylogenetic analysis, it has several problems. The resolution of 16S rRNA gene sequence analysis between closely related species is generally low [15] and lateral gene transfer of rDNA were also reported [51], [52]. In addition, rDNAs have limited information due to their short sequence. The present method at least partly overcome these problems and is potentially more reliable tool than 16S rDNA and other single gene to infer the evolutionary relationships of organisms.

The present method showed advantages in that a reliable phylogenetic tree could be constructed, even though the organisms compared have a small number of conserved (core) genes (Figs. 3, 4, S1 and S2). This feature makes the present method applicable to a wide range of organisms, including those for which only partial genome sequences are available. Improvement of our whole-genome-based method, as well as progress in theoretical considerations, will contribute to a genome-based understanding especially of the bacterial phylogeny.

## Supporting Information

Figure S1 **Consensus phylogenetic trees of** ***Prochlorococcus*** **and** ***Synechococcus*** **species constructed using the reduced gene content of** ***Synechococcus*** **sp. WH8102.** Procedures for the construction of consensus phylogenetic trees are the same as used in Fig. 3. Ten independent databases of *Synechococcus* sp. WH8102 were artificially formed with 253 randomly selected genes (10% of the total gene number). *S. elongatus* PCC 6301 was used as an out-group.
(PDF)

Figure S2 **Phylogenetic tree of small number of photosynthetic prokaryotes.** Procedures for the construction of the phylogenetic tree are the same as in Figs. 2B and 4. Alphabetical characters (A-AY) represent the branch points. Numbers on the branch points are the bootstrap values for each node. Bootstrap values were obtained from 100 reproduced trees of 1,000 randomly selected E-values. Archaea were used as an out-group.
(PDF)

Figure S3 **Phylogenetic tree based on the reciprocal best BLAST hits.** Procedures and protein sequence databases used for the construction of phylogenetic tree are the same as in Fig. S2 except that distances and bootstrap values were estimated from the E-values of reciprocal best BLAST hits. Bootstrap values were obtained from 100 reproduced trees of 1,000 randomly selected E-values of reciprocal best BLAST hits. Archaea were used as an out-group.
(PDF)

Figure S4 **Comparison of E-values of the best-matched proteins of photosynthetic bacteria.** *Synechocystis* proteins of (a) PS I, (b) PS II, (c) cyt *b*_6_/*f* and electron career proteins, (d) CO_2_ concentration and assimilation, and (e) chlorophyll biosynthesis were used as the query for BLAST search against the database of *Heliobacterium modesticaldum* (heliobacteria), a merged database of *Chlorobium*, *Pelodictyon*, and *Prosthecochloris* species (green sulfur bacteria), a merged database of *Rhodobacter*, *Roseobacter*, *Rhodopseudomonas*, and *Rhodospirillum* species (purple bacteria), and a merged database of *Roseiflexus* and *Chloroflexu*s species (green filamentous bacteria). E-values of the each *Synechocystis* protein against best-matched proteins of these four hypothetical databases of photosynthetic bacteria were plotted. Diamonds, green filamentous bacteria; squares, green sulfur bacteria; triangles, heliobacteria; circles, purple bacteria.
(PDF)

Table S1 **List of 16S rDNA genes used for the calculation of substitution rates of 16S rDNA sequences and construction of the phylogenetic tree in** **Fig. 2A** **.**
(XLS)

Table S2 **List of bacterial genome databases used for phylogenetic analysis.**
(XLS)

Table S3 **Gene content and genome sizes of** ***Prochlorococcus*** **and** ***Synechococcus*** **.**
(XLS)

Table S4 **Distance matrix of photosynthetic bacteria and archaea.**
(XLS)
