# Supplementary material for: Construction of a Phylogenetic Tree of Photosynthetic Prokaryotes Based on Average Similarities of Whole Genome Sequences
Source: PLoS One. 2013 Jul 26;8(7):e70290. doi: 10.1371/journal.pone.0070290 (PMC3724816; doi:10.1371/journal.pone.0070290)
Supplement: Figure S1 — Consensus phylogenetic trees of Prochlorococcus and Synechococcus species constructed using the reduced gene content of Synechococcus sp. WH8102. Procedures for the construction of consensus phylogenetic trees are the same as used in Fig. 3. Ten independent databases of Synechococcus sp. WH8102 were artificially formed with 253 randomly selected genes (10% of the total gene number). S. elongatus PCC 6301 was used as an out-group. (PDF) [file pone.0070290.s001.pdf]

# Fig. S1

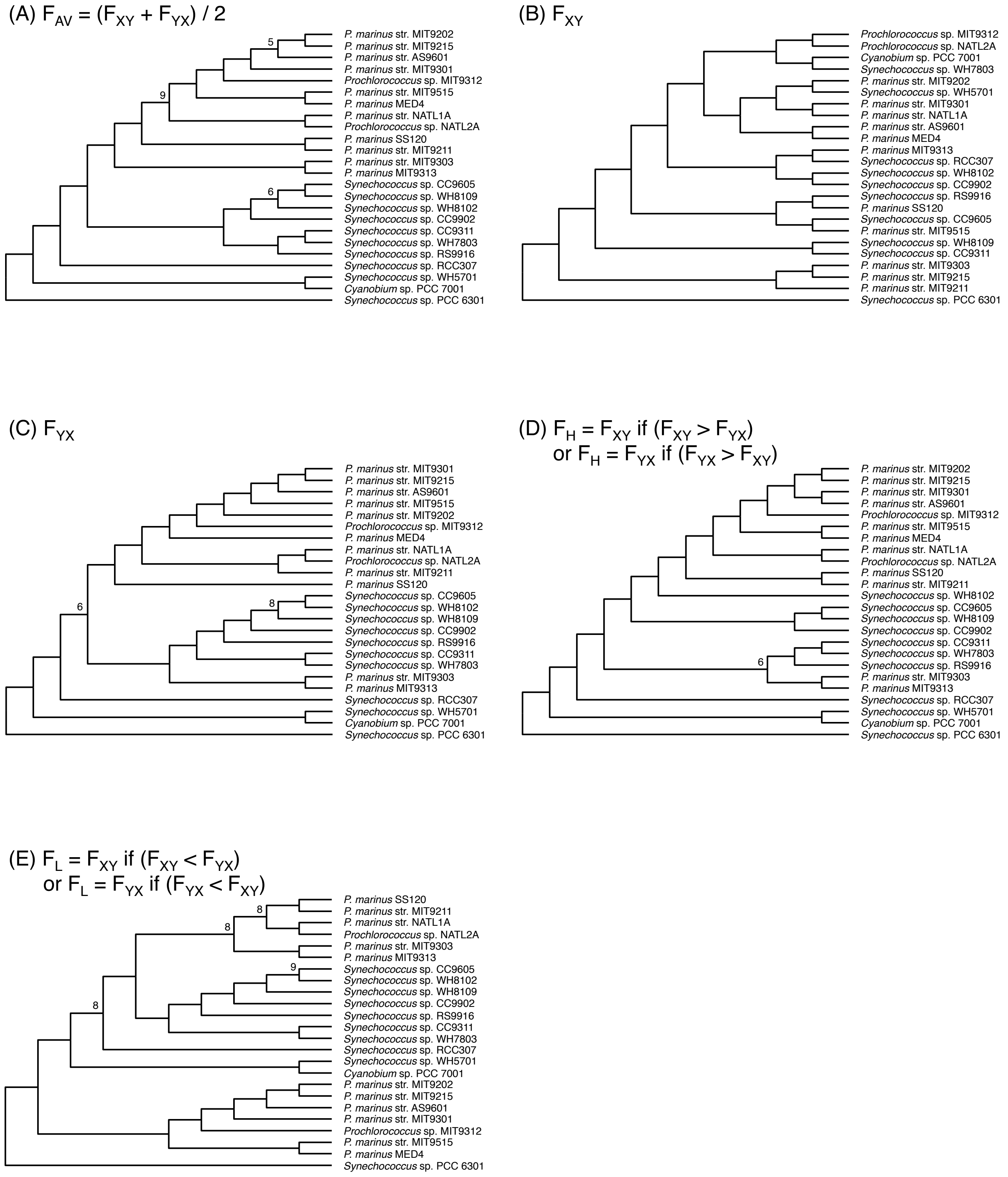


Satoh et al.
