# Supplementary figures and images for: Construction of a Phylogenetic Tree of Photosynthetic Prokaryotes Based on Average Similarities of Whole Genome Sequences
Source: PLoS One. 2013 Jul 26;8(7):e70290. doi: 10.1371/journal.pone.0070290 (PMC3724816; doi:10.1371/journal.pone.0070290)

Figure S2

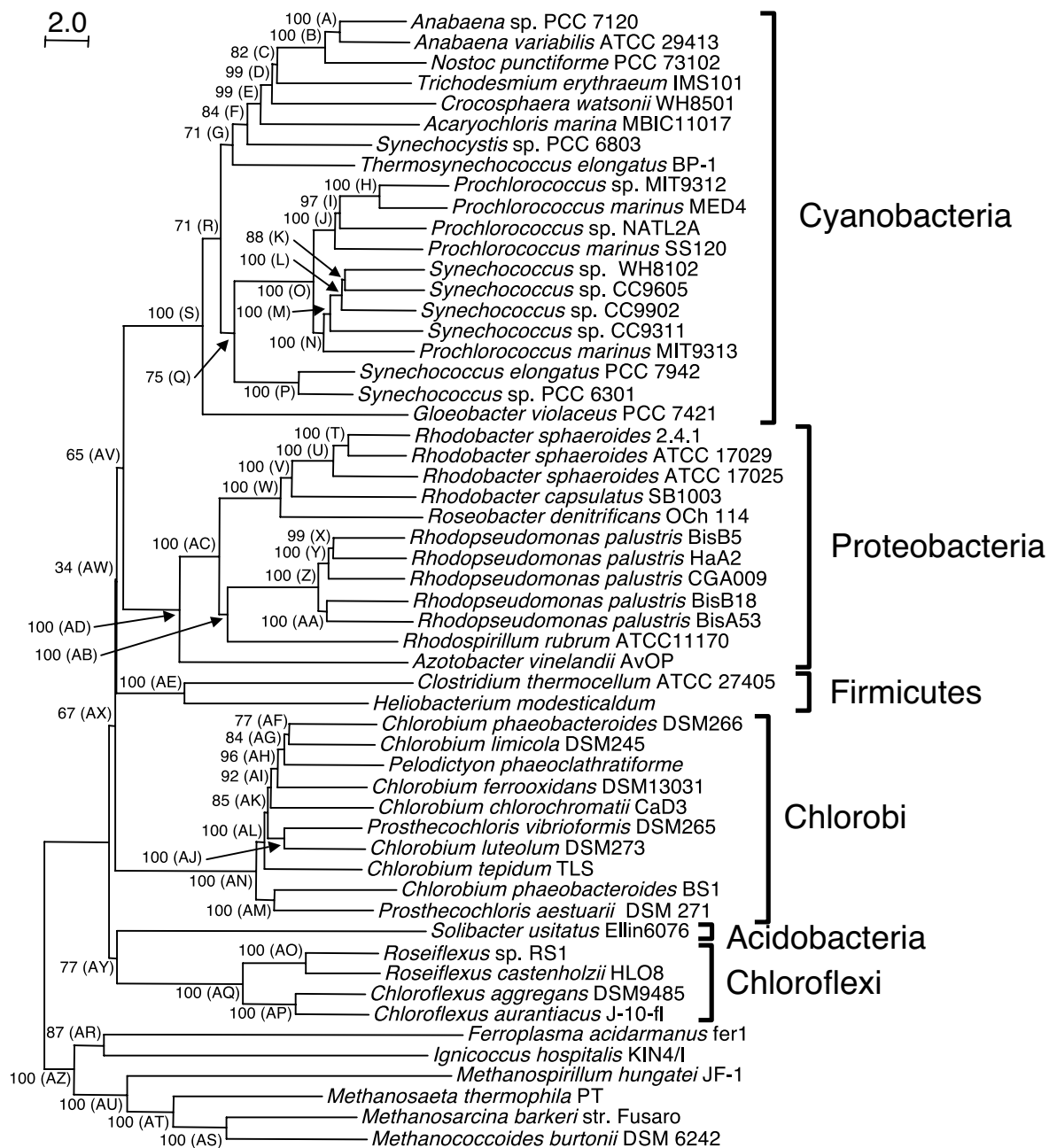

Supplement: Figure S2 — Phylogenetic tree of small number of photosynthetic prokaryotes. Procedures for the construction of the phylogenetic tree are the same as in Figs. 2B and 4. Alphabetical characters (A-AY) represent the branch points. Numbers on the branch points are the bootstrap values for each node. Bootstrap values were obtained from 100 reproduced trees of 1,000 randomly selected E-values. Archaea were used as an out-group. (PDF) [file pone.0070290.s002.pdf]

Figure S3

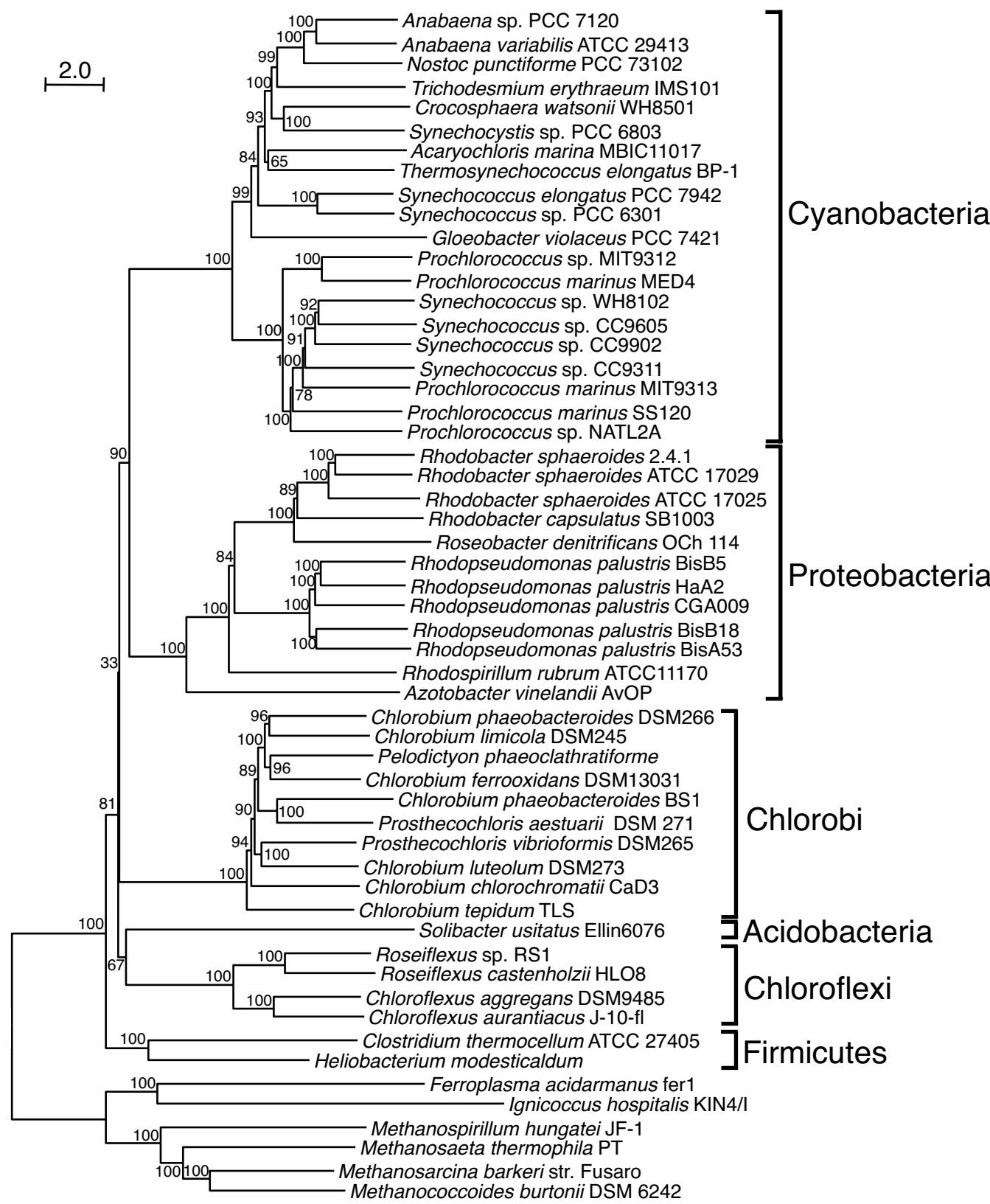

Supplement: Figure S3 — Phylogenetic tree based on the reciprocal best BLAST hits. Procedures and protein sequence databases used for the construction of phylogenetic tree are the same as in Fig. S2 except that distances and bootstrap values were estimated from the E-values of reciprocal best BLAST hits. Bootstrap values were obtained from 100 reproduced trees of 1,000 randomly selected E-values of reciprocal best BLAST hits. Archaea were used as an out-group. (PDF) [file pone.0070290.s003.pdf]
