# Supplementary material for: Construction of a Phylogenetic Tree of Photosynthetic Prokaryotes Based on Average Similarities of Whole Genome Sequences
Source: PLoS One. 2013 Jul 26;8(7):e70290. doi: 10.1371/journal.pone.0070290 (PMC3724816; doi:10.1371/journal.pone.0070290)
Supplement: Figure S4 — Comparison of E-values of the best-matched proteins of photosynthetic bacteria. Synechocystis proteins of (a) PS I, (b) PS II, (c) cyt b 6/f and electron career proteins, (d) CO2 concentration and assimilation, and (e) chlorophyll biosynthesis were used as the query for BLAST search against the database of Heliobacterium modesticaldum (heliobacteria), a merged database of Chlorobium, Pelodictyon, and Prosthecochloris species (green sulfur bacteria), a merged database of Rhodobacter, Roseobacter, Rhodopseudomonas, and Rhodospirillum species (purple bacteria), and a merged database of Roseiflexus and Chloroflexus species (green filamentous bacteria). E-values of the each Synechocystis protein against best-matched proteins of these four hypothetical databases of photosynthetic bacteria were plotted. Diamonds, green filamentous bacteria; squares, green sulfur bacteria; triangles, heliobacteria; circles, purple bacteria. (PDF) [file pone.0070290.s004.pdf]

Figure S4

a) - PSI proteins

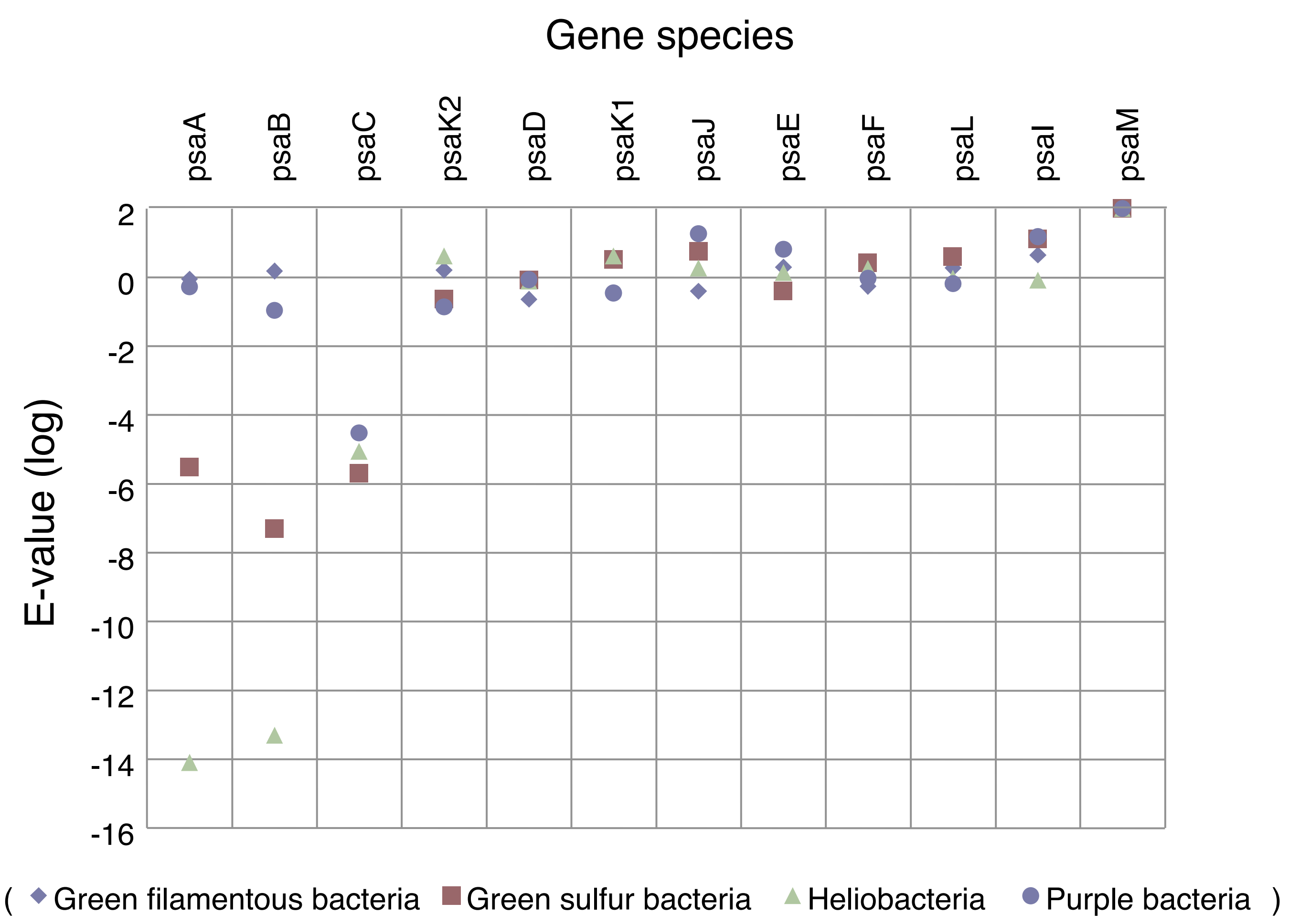

Satoh et al.

b) - PSII proteins

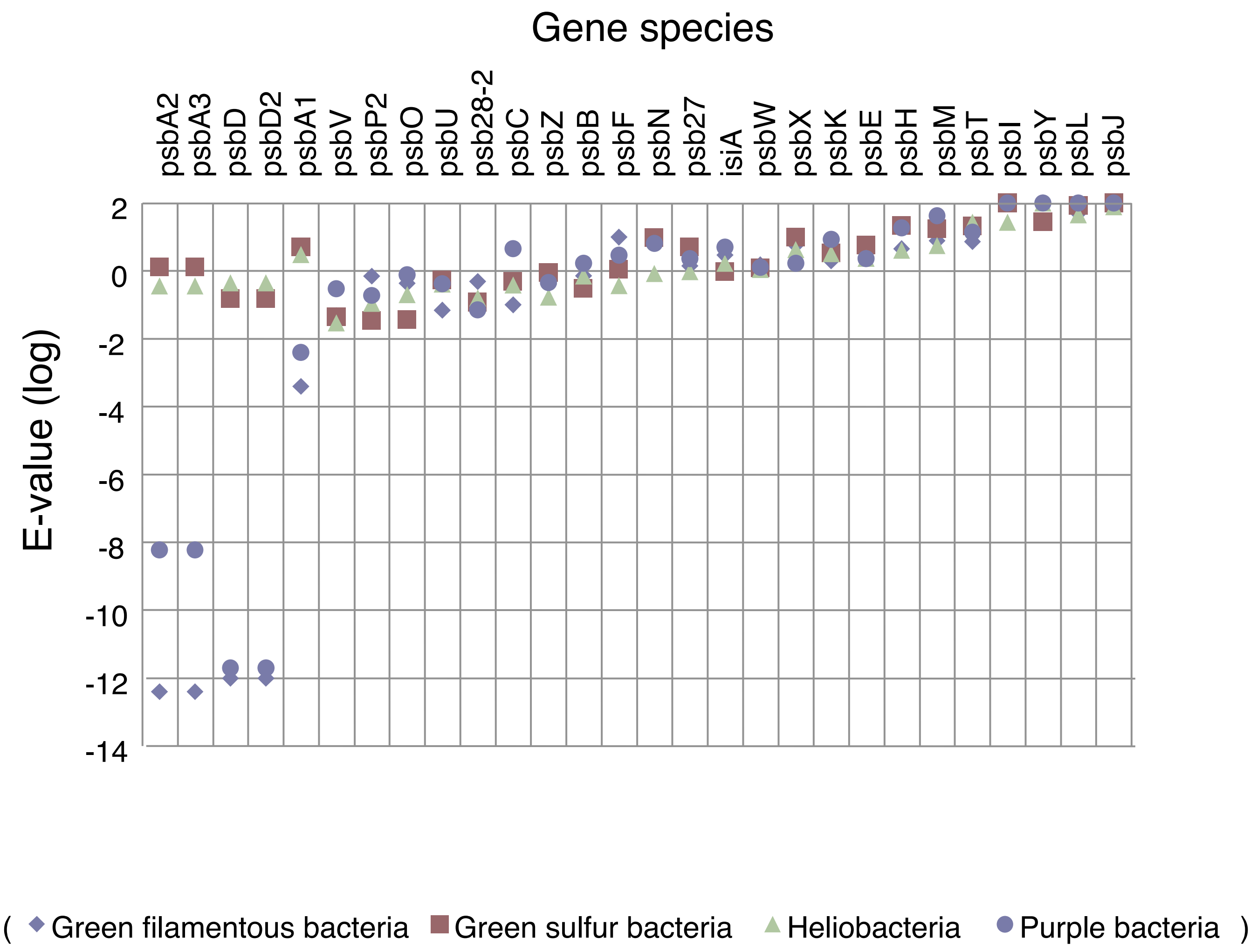

Satoh et al.

c) - Cytochrome b_6f proteins and electron carrier proteins

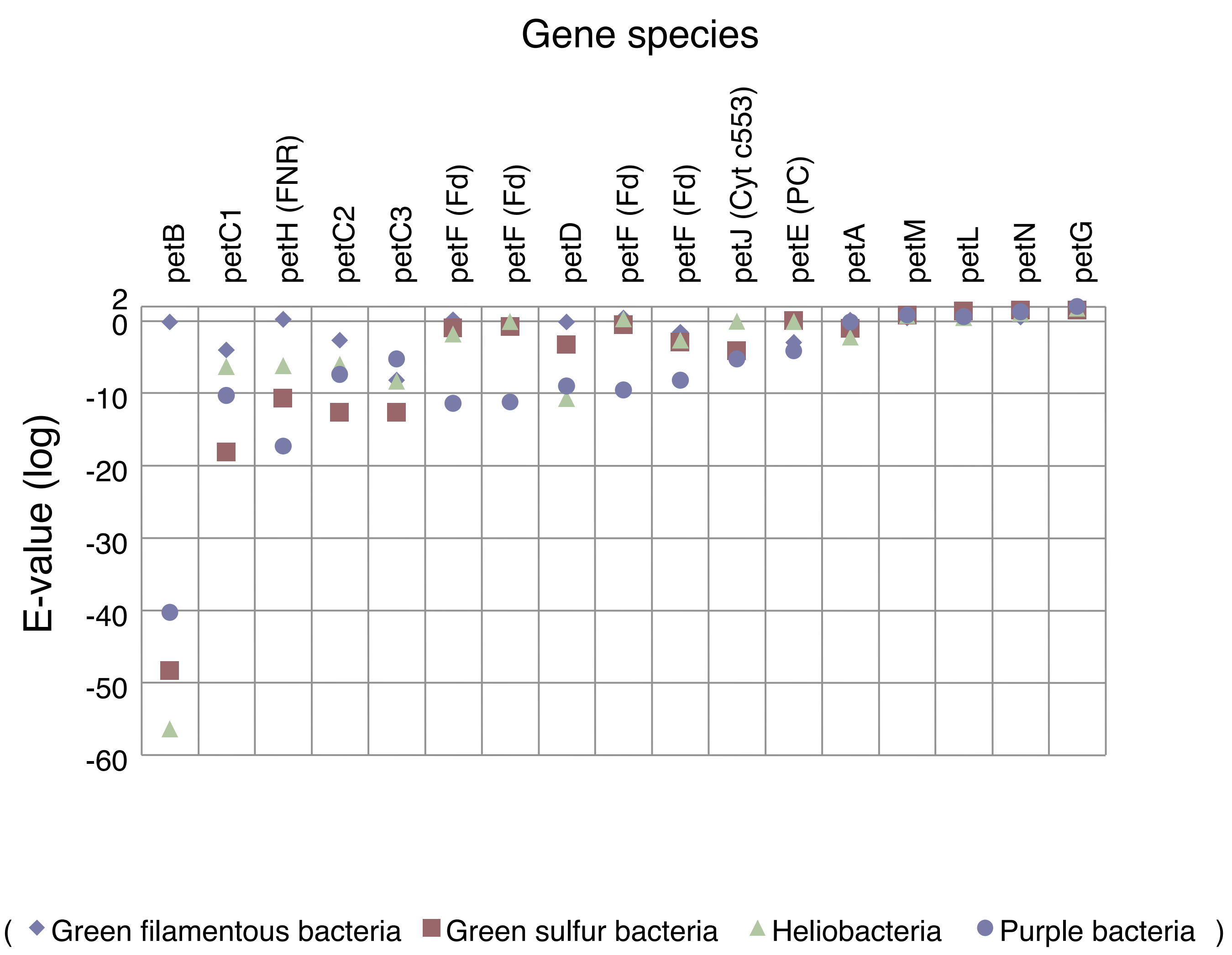

Satoh et al.

d) - Carbon concentration and assimilation

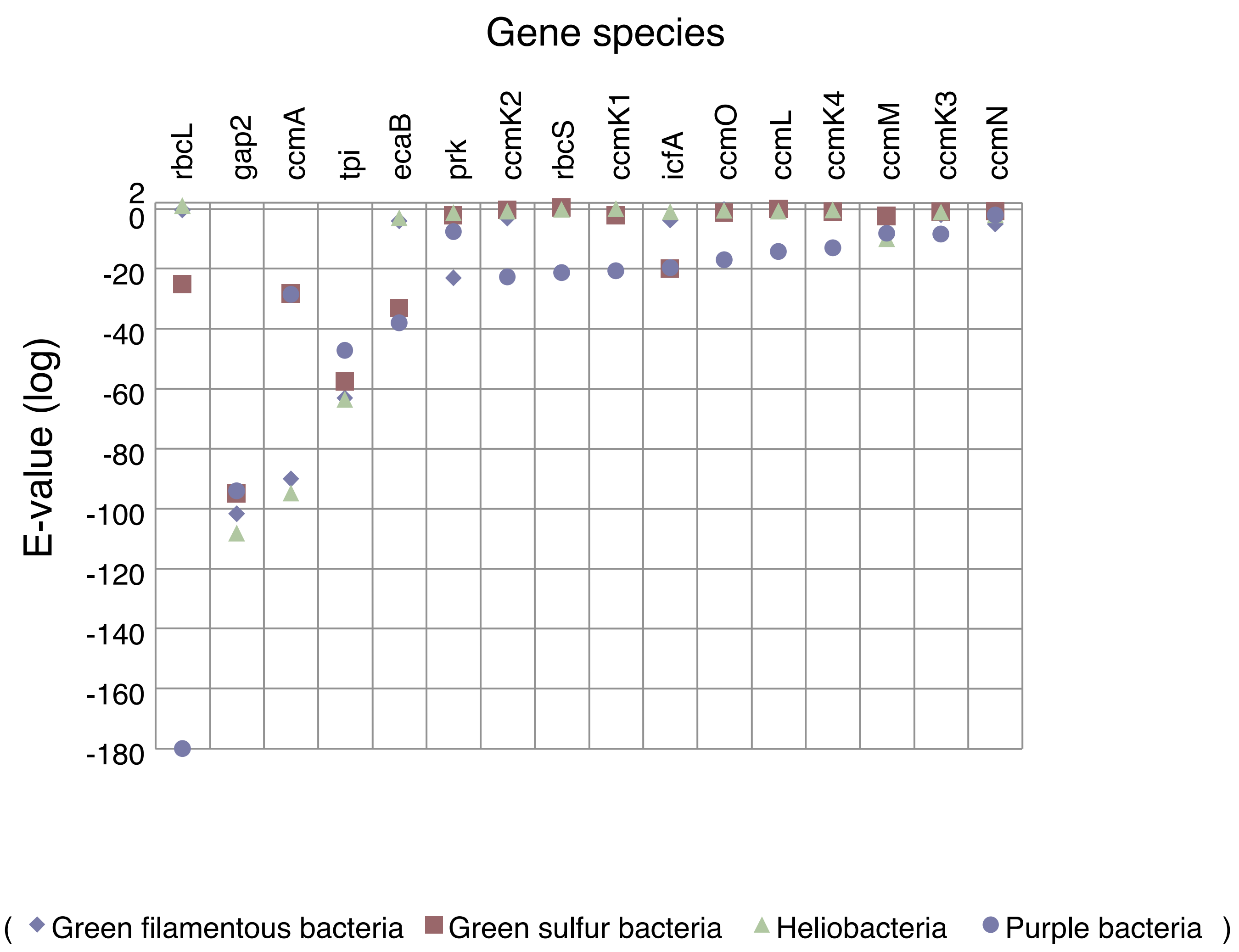

Satoh et al.

e) - Chlorophyll biosynthesis

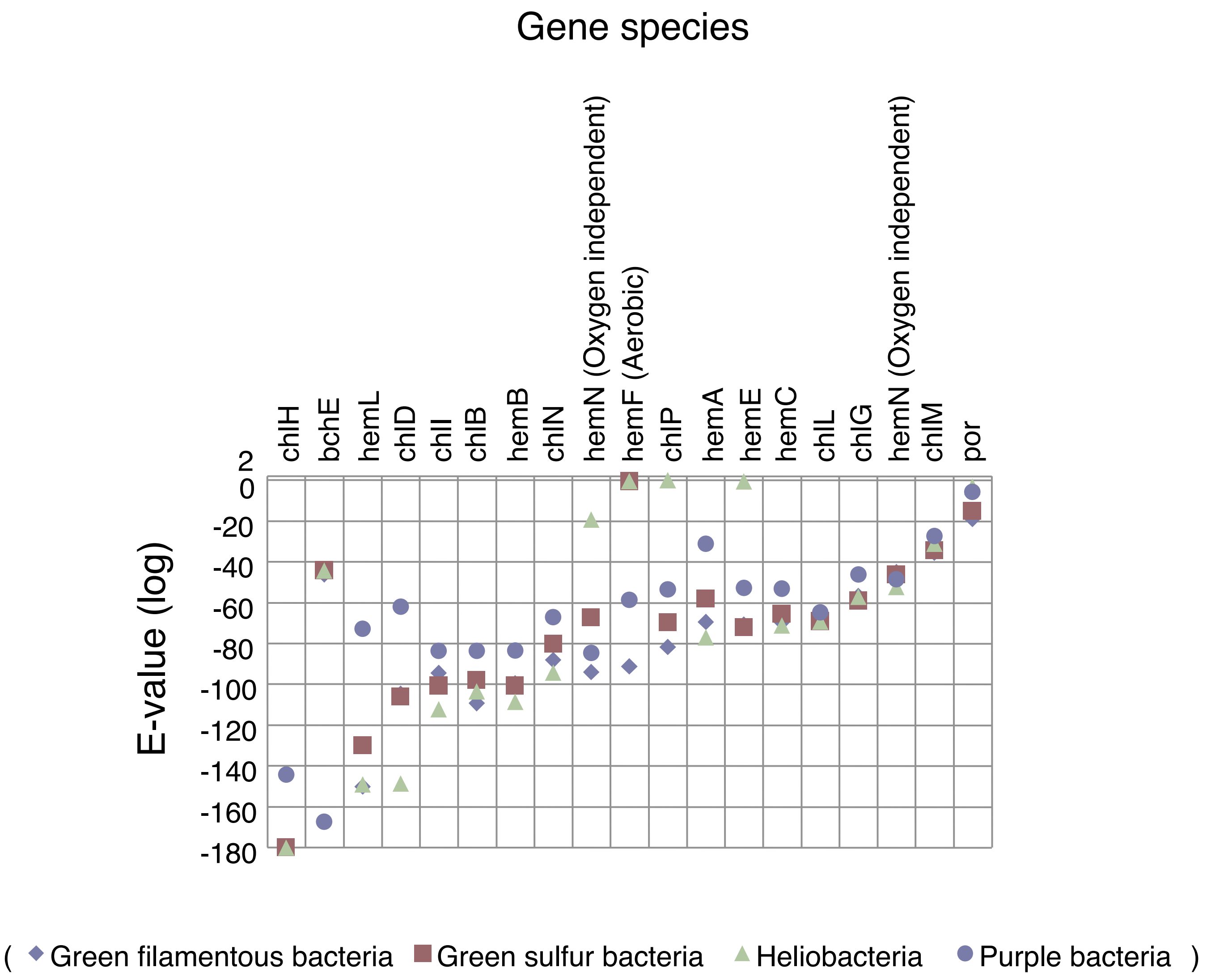

Satoh et al.
